# *GSTM1*, *GSTT1*, and *GSTP1* Polymorphisms and Associations between Air Pollutants and Markers of Insulin Resistance in Elderly Koreans

**DOI:** 10.1289/ehp.1104406

**Published:** 2012-06-25

**Authors:** Jin Hee Kim, Yun-Chul Hong

**Affiliations:** 1 Institute of Environmental Medicine, Seoul National University Medical Research Center, Seoul, Republic of Korea; 2 Department of Preventive Medicine, Seoul National University College of Medicine, Seoul, Republic of Korea

**Keywords:** air pollution, elderly, genetic polymorphism, insulin resistance

## Abstract

Background: Previous studies have suggested that diabetes mellitus (DM) is an outcome of exposure to air pollution, and metabolic detoxification genes affect air pollution–related outcomes.

Objectives: We evaluated associations between air pollutants and markers of insulin resistance (IR), an underlying mechanism of type 2 DM, and effect modification by *GSTM1, GSTT1,* and *GSTP1* genotypes among elderly participants in the Korean Elderly Environmental Panel (KEEP) study.

Methods: We recruited 560 people ≥ 60 years of age and obtained blood samples from them up to three times between 2008 and 2010. For air pollution exposure, we used ambient air pollutant [i.e., particulate matter ≤ 10 µm in diameter (PM_10_), sulfur dioxide (SO_2_), ozone (O_3_), and nitrogen dioxide (NO_2_)] monitoring data. We measured levels of fasting glucose and insulin and derived the homeostatic model assessment (HOMA) index to assess IR. Mixed-effect models were used to estimate associations between air pollutants and IR indices on the same day or lagged up to 10 days prior, and effect modification by *GSTM1, GSTT1,* and *GSTP1* genotypes.

Results: Interquartile range increases in PM_10_, O_3_, and NO_2_ were significantly associated with IR indices, depending on the lag period. Associations were stronger among participants with a history of DM and among those with *GSTM1*-null, *GSTT1*-null, and *GSTP1* AG or GG genotypes.

Conclusions: Our results suggest that PM_10_, O_3_, and NO_2_ may increase IR in the elderly, and that *GSTM1*-null, *GSTT1*-null, and *GSTP1* AG or GG genotypes may increase susceptibility to potential effects of ambient air pollutants on IR.

Insulin resistance (IR) has been regarded as an important health issue because it affects the development of type 2 diabetes mellitus (DM) (Grattagliano et al. 2008; Marx 2002). The number of patients with DM has grown rapidly and is expected to continue to increase worldwide (Hoang et al. 2007; Marx 2002; Zarich 2006).

Recently, exposure to nitrogen dioxide (NO_2_) and proximity to roads (which serves as a proxy for traffic-related pollutant exposure) have been associated with DM (Brook et al. 2008; Puett et al. 2011). This result raised the possibility that DM is affected directly by air pollution exposure in addition to being an effect modifier for air pollution–associated diseases (Bateson and Schwartz 2004; Dubowsky et al. 2006; Hathout et al. 2002; O’Neill et al. 2005; Peel et al. 2007; Zanobetti and Schwartz 2001). Correlations between air pollution and markers of IR have also been reported in a cross-sectional study of children (Kelishadi et al. 2009).

Recent research has suggested that oxidative stress is a major biologic pathophysiological mechanism underlying the adverse health effects of air pollutants (Romieu et al. 2010). Therefore, genes involved in oxidative stress are logical candidates for studying air pollution × gene interactions. The human glutathione *S*-transferase genes are well-known oxidative stress–related detoxification enzymes. Deletions of the glutathione *S*-transferase M1 (*GSTM1)* and T1 (*GSTT1*) genes lead to null phenotypes completely lacking enzyme function. A polymorphic site at codon 105 (A to G substitution, resulting in a change of isoleucine to valine) of *GST* P1 alters enzyme-binding kinetics for some electrophilic substrates (Zimniak et al. 1994). We hypothesized that *GSTM1*, *GSTT1*, and *GSTP1* polymorphisms may contribute to susceptibility to air pollution–related outcomes.

We conducted a longitudinal panel study of elderly Koreans to estimate the effects of air pollutants on fasting blood levels of glucose and insulin and the homeostatic model assessment (HOMA) index of IR, and evaluated effect modification by *GSTM1*, *GSTT1,* and *GSTP1* genotypes. We obtained air monitoring data from the Korea National Institute of Environmental Research.

## Materials and Methods

*Study population and sampling.* The Korean Elderly Environmental Panel (KEEP) study was launched in March 2008 to explore relationships between environmental exposures and health outcomes in the elderly. From its start to 2010, the KEEP study recruited a total of 560 persons ≥ 60 years of age at their first visit. Participants completed a medical examination at a community elderly welfare center in the Seongbuk-Gu area of Seoul, Korea, up to five times during the study period (twice in 2008, once in 2009, and twice in 2010), and provided up to three fasting blood samples (≤ 1/year). All serum samples were placed at –70°C immediately after collection and stored until analyzed for glucose and insulin. Trained interviewers also obtained detailed information from participants using a structured questionnaire including demographics, lifestyle habits, and medical history. The study protocol was approved by the institutional review board at Seoul National University Hospital, Seoul, Republic of Korea (ROK) and each study participant provided written informed consent.

*Air pollution concentrations and outdoor temperature.* Ambient air pollutant [i.e., particulate matter ≤ 10 µm in diameter (PM_10_), sulfur dioxide (SO_2_), ozone (O_3_), and NO_2_] concentration data were obtained from the Korea National Institute of Environmental Research, Incheon, ROK. Air pollutant concentrations measured at the monitoring center nearest to the residence of each subject were used to estimate individual exposures to ambient pollutants. The average distance between monitoring sites and individual residences was < 1 km. Air pollutant exposure measures were computed as daily mean concentrations on the day of the study visit (lag 0) and on each of the 10 days before the visit (lag 1 through lag 10). Daily average outdoor temperature and dew point data measured at the Songwol-dong monitoring center nearest to each participant’s residence were obtained from the Korea Meteorological Administration, Seoul, ROK.

*Glucose and insulin.* IR is characterized by elevated serum insulin concentrations in association with a normal or high fasting glucose concentration in serum. Therefore, we measured fasting glucose and insulin levels in serum collected on health examination days to evaluate IR and impaired glucose tolerance. Briefly, glucose levels were determined by the hexokinase method using a Pureauto S GLU kit (Daiichi Pure Chemicals, Tokyo, Japan), and insulin levels were determined by radioimmunoassay with the double-antibody batch method using Elecsys Insulin and Elecsys 2010 Immunoanalyser (both from Roche Diagnostics, Mannheim, Germany). We also calculated the HOMA as an index of IR according to the following equation: fasting insulin (in microunits per milliliter) × [fasting glucose (in millimoles per liter) ÷ 22.5] (Matthews et al. 1985).

*Cotinine.* Urinary cotinine levels for monitoring tobacco exposure were measured in samples obtained during the same study visits as the fasting blood samples. Cotinine levels were analyzed by an enzyme-linked immunosorbent assay (ELISA) method based upon the competitive binding to antibody of enzyme labeled antigen and unlabeled antigen (Cotinine Elisa; Bio-Quant, San Diego, CA, USA) following the manufacturer’s recommended procedure.

GSTM1, GSTT1, *and* GSTP1 *genotyping.* Genomic DNA was extracted from peripheral blood lymphocytes using a QIAamp DNA Blood Mini Kit (Qiagen, Valencia, CA, USA), and genetic polymorphisms of *GSTM1*, *GSTT1*, and *GSTP1* were determined using a multiplex polymerase chain reaction method (Kim et al. 2006). Identical results were obtained when genotyping was repeated in a 10% random sample.

*GSTP1* polymorphism (rs1695) frequencies of 0.66 (*n* = 359), 0.31 (*n* = 171), and 0.03 (*n* = 18) for the AA, AG, and GG genotypes were consistent with the Hardy–Weinberg equilibrium (*p* > 0.05 by chi-square test).

*Statistical analysis.* We estimated the effects of each pollutant on glucose, insulin, and HOMA indices using individual linear mixed-effect models for repeated measures analysis after adjusting for age, sex, body mass index [BMI; weight (in kilograms) ÷ height (in meters squared)], cotinine level, and outdoor temperature and dew point of the day. All covariates other than sex were modeled as continuous variables. In addition, we fit two- and three-pollutant models to assess potential confounding by co-pollutants. We estimated delayed effects of air pollutants on IR indices using individual daily average lag structures up to 10 days before the health examination, and estimated accumulated effects of air pollutants over multiday lag periods (0–1 days, 0–2 days, . . . 0–10 days) using an unconstrained distributed lag model. Because IR is a risk factor for DM, the effects of each air pollutant on IR indices were estimated separately according to DM history.

Associations between air pollutants and glucose, insulin, and HOMA indices were also estimated according to *GSTM1*, *GSTT1*, and *GSTP1* genotypes. To ascertain the interactions between gene and air pollution on IR indices, interaction *p*-values for term air pollutants × genotypes were estimated in the same model. A penalized regression spline of exposure to air pollutants on glucose, insulin, and HOMA indices by genotypes was evaluated using generalized additive mixed models to ascertain whether associations between air pollutants and IR indices according to genotypes were linear.

The number of repeated measurements varied among participants, which may have led to selection bias if the loss to follow-up was not random (Rubin 1976). Therefore, we conducted all analyses after weighting follow-up observations by the inverse probability of attaining a follow-up response (Robins et al. 1995). For participants who completed > 1 visit, we used logistic regression to predict the probability of a follow-up visit (follow-up = 1, missing = 0) according to age, sex, BMI, number of years of schooling, blood pressure, season, and outdoor temperature at the prior visit. The first observation for each participant was given a weight of 1, and more weight (the inverse of the predicted probability of having a follow-up response) was assigned to subsequent observations that were more likely missing.

SAS version 9.2 (SAS Institute Inc., Cary, NC, USA) and R version 2.12.1 [Comprehensive R Archive Network (http://cran.r-project.org)] were used for statistical analyses. Alpha level for statistical significance was 0.05.

## Results

At baseline, there was a total of 560 participants ≥ 60 years of age, of whom 146 (26.1%) were male and 414 (73.9%) were female (Table 1). The mean number of visits was 3.3, with more visits among females than males (3.4 vs. 3.1 visits, *p* = 0.04). BMI and serum insulin levels were significantly higher in females than males (*p* = 0.02 and *p* = 0.047, respectively). A history of DM, hypertension, or hyperlipidemia was reported by 91 (16.3%), 285 (50.9%), and 183 (32.7%) participants, respectively. Blood samples for genotyping were available for 548 participants.

**Table 1:** Baseline characteristics of participants by sex.

Characteristic	Male	Female	Total
Participants [n (%)]	146 (26.1)	414 (73.9)	560 (100)
Visits (mean ± SD)	3.1 ± 1.3	3.4 ± 1.4	3.3 ± 1.4
Age [mean (range)]	71.4 (62–84)	70.5 (60–87)	70.7 (60–87)
Height (cm; mean ± SD)	164.3 ± 5.3	151.3 ± 5.1	154.7 ± 5.1
Weight (kg; mean ± SD)	65.7 ± 9.8	57.1 ± 7.4	59.3 ± 8.1
BMI [kg/m2; n (%)]
≥ 30	5 (3.4)	19 (4.6)	24 (4.3)
25 to < 30	51 (34.9)	168 (45.2)	219 (39.1)
< 25	90 (61.7)	227 (54.8)	317 (56.6)
Current smokers [n (%)]	31 (21.2)	1 (0.2)	32 (5.7)
Glucose (fasting levels in serum)	5.42 ± 1.15	5.32 ± 1.13	5.34 ± 1.14
[mmol/L; mean ± SD (range)]	(3.50 – 10.99)	(3.89 – 16.32)	(3.50 – 16.32)
Insulin (fasting levels in serum)	6.15 ± 4.58	7.14 ± 6.41	6.88 ± 5.99
[μU/mL; mean ± SD (range)]	(0.70 – 28.00)	(0.90 – 76.30)	(0.70 – 76.30)
HOMA	1.54 ± 1.31	1.76 ± 1.84	1.70 ± 1.72
[mean ± SD (range)]	(0.15 – 7.39)	(0.22 – 21.46)	(0.15 – 21.46)
Disease history [n (%)]
DM	25 (4.5)	66 (11.8)	91 (16.3)
Hypertension	76 (52.1)	209 (50.5)	285 (50.9)
Hyperlipidemia	47 (32.2)	136 (32.9)	183 (32.7)
Participants for whom DNA samples were obtained [n (%)]	142 (97.3)	406 (98.1)	548 (97.9)
GSTM1
Present	64 (45.1)	172 (42.4)	236 (43.1)
Null	78 (54.9)	234 (57.6)	312 (56.9)
GSTT1
Present	72 (50.7)	193 (47.5)	265 (48.4)
Null	70 (49.3)	213 (52.5)	283 (51.6)
GSTP1 (rs1695)
AA	90 (63.4)	269 (66.3)	359 (65.5)
AG	47 (33.1)	124 (30.5)	171 (31.2)
GG	5 (3.5)	13 (3.2)	18 (3.3)
The HOMA index uses the formula described by Matthews et al. (1985): insulin (μU/mL) × [glucose (mmol/L) ÷ 22.5].						

Participants completed a total of 1,850 individual health examinations. We estimated each participant’s exposures to air pollutants using air monitoring data for the visit day and the 10 days prior to each health examination during the study period (Table 2). Exposures to PM_10_, SO_2_, O_3_, and NO_2_ averaged over the visit day through lag day 10 were 42.58 µg/m^3^, 3.80 ppb, 19.38 ppb, and 35.13 ppb, respectively. Mean outdoor temperature and dew point values on the health examination days were 17.24°C and 6.78°C, respectively. Average exposures on each lag day were similar to average exposures on other lag days [see Supplemental Material, Figure S1 (http://dx.doi.org/10.1289/ehp.1104406)] and average air pollutant levels on health examination days were correlated (all *p*-values < 0.0001) (see Supplemental Material, Table S1).

**Table 2:** Distribution of air pollutant levels over 11 days, including each health examination day.

Air pollutant	*n*	Mean ± SD	Selected percentiles										
			10th	25th	50th	75th	90th	95th
PM10 (µg/m3)	1,850	42.58 ± 16.81	23.08	29.92	39.94	50.75	68.12	79.51
SO2 (ppb)	1,850	3.80 ± 1.45	2.43	2.72	3.27	4.35	5.99	6.94
O3 (ppb)	1,850	19.38 ± 7.96	8.99	11.58	19.34	26.67	29.56	31.33
NO2 (ppb)	1,850	35.13 ± 8.46	24.09	28.51	35.23	39.30	45.78	48.16
Outdoor temperature (°C)	1,906	17.24 ± 8.57	3.73	11.27	18.62	24.87	26.48	26.75
Dew point (°C)	1,906	6.78 ± 10.19	–7.58	–1.91	7.45	16.73	19.52	20.04
Exposures to air pollutants and meteorological elements were averaged over the visit day through lag day 10.																

**Figure 1:**
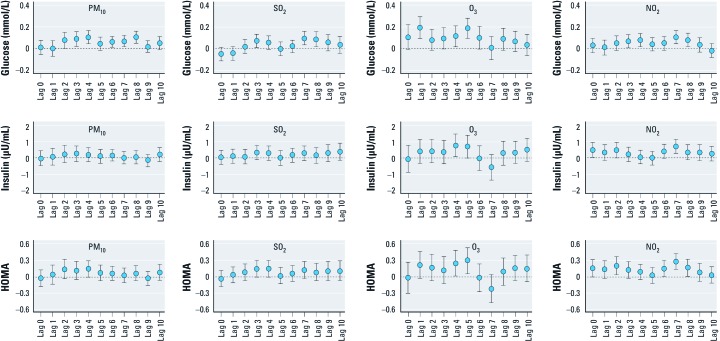
Associations of PM_10_, SO_2_, O_3_, and NO_2_ with glucose, insulin, and HOMA indices according to lag day. Changes in glucose, insulin, and HOMA indices by an IQR change of PM_10_ (20.8 µg/m^3^), SO_2_ (1.6 ppb), O_3_ (15.1 ppb), and NO_2_ (10.8 ppb) were estimated in linear mixed-effect models after weighting follow-up observations and adjusting for age, sex, BMI, cotinine level, and outdoor temperature and dew point of the day. For details, see Supplemental Material, Table S2, (http://dx.doi.org/10.1289/ehp.1104406).

We used linear mixed-effect models to estimate the effects of PM_10_, SO_2_, O_3_, and NO_2_ on glucose, insulin, and HOMA indices for each individual lag day after adjusting for age, sex, BMI, cotinine level, outdoor temperature, and dew point, with weights applied to account for loss to follow-up. Significant associations with HOMA were observed for interquartile range (IQR) increases in PM_10_, O_3_, and NO_2_, with the strongest associations observed on lag day 4 for PM_10_ [0.14 increase in HOMA; 95% confidence interval (CI): –0.003, 0.29; *p*-value = 0.05], lag day 5 for O_3_ (0.30; CI: 0.06, 0.53; *p*-value = 0.01), and lag day 7 for NO_2_ (0.28; CI: 0.13, 0.42; *p*-value = 0.0002) [Figure 1; see also Supplemental Material, Table S2 (http://dx.doi.org/10.1289/ehp.1104406)]. Glucose and insulin showed similar trends with HOMA except for no association between PM_10_ and insulin. SO_2_ was not significantly associated with the HOMA and insulin indices for any lag, but was significantly associated with fasting glucose on lag days 3, 7, and 8. Even though effect estimates for SO_2_ and HOMA and insulin look very similar to those for PM_10_, the relationship between SO_2_ and HOMA was not significant for any lag. Therefore, we further analyzed only for PM_10_, O_3_, and NO_2_.

Multiple-pollutant models were generally consistent with single-pollutant models. Estimates for NO_2_ in the three-pollutant model appeared similar with those in the single-pollutant model (0.07 for glucose; CI: 0.01, 0.14; 0.67 for insulin; CI: 0.23, 1.11; and 0.24 for HOMA; CI: 0.09, 0.39). Estimates for NO_2_ in the two-pollutant model also appear similar with those in the single-pollutant model and in the three-pollutant model (all *p*-values < 0.01). PM_10_ in the two-pollutant model showed consistent and significant association with glucose level with marginal significance in the three-pollutant model although there was no association with insulin or HOMA (0.08 after controlling for O_3_; CI: 0.01, 0.14; 0.08 after controlling for NO_2_; CI: 0.02, 0.15; and 0.06 after controlling for O_3_ and NO_2_; CI: –0.005, 0.12), and O_3_ showed at least marginally significant association with all IR indices after controlling for PM_10_ (0.15 for glucose; CI: 0.05, 0.25; 0.67 for insulin; CI: –0.06, 1.39; and 0.25 for HOMA; CI: –0.001, 0.49) [see Supplemental Material, Table S3 (http://dx.doi.org/10.1289/ehp.1104406)]. Accumulated effects of IQR increases in pollutants estimated using a distributed lag model were strongest for lag 0–10 for PM_10_ (0.25 for HOMA; CI: –0.49, 0.99), lag 0–5 for O_3_ (0.35 for HOMA; CI: –0.36, 1.07), and lag 0–8 for NO_2_ (0.61 for HOMA; CI: 0.03, 1.18), although associations were not statistically significant for PM_10_ and O_3_, (see Supplemental Material, Figure S2). SO_2_ did not appear to be associated with the three IR indices based on distributed lag models.

Associations were estimated separately among participants without and with a history of DM (Table 3). Associations were more apparent among participants with a history of DM.

**Table 3:** Estimated associations for exposure to PM_10_, O_3_, and NO_2_ with fasting glucose, insulin, and HOMA indices in subjects without and with a history of DM.

Pollutant	Glucose				Insulin				HOMA			
	Estimate (95% CI)		*p*-Value	Estimate (95% CI)		*p*-Value	Estimate (95% CI)		*p*-Value
Total (n = 560)
PM10	0.11	(0.05, 0.17)	0.0005	0.21	(–0.22, 0.64)	0.3439	0.14	(–0.003, 0.29)	0.0549
O3	0.19	(0.09, 0.28)	0.0001	0.71	(0.02, 1.39)	0.0439	0.30	(0.06, 0.53)	0.0139
NO2	0.11	(0.05, 0.17)	0.0004	0.72	(0.29, 1.14)	0.0010	0.28	(0.13, 0.42)	0.0002
Without history of DM (n = 469)
PM10	0.05	(0.01, 0.10)	0.0112	–0.14	(–0.58, 0.29)	0.5198	–0.03	(–0.17, 0.11)	0.7102
O3	0.09	(0.02, 0.16)	0.0080	0.32	(–0.39, 1.03)	0.3766	0.12	(–0.11, 0.35)	0.3097
NO2	0.05	(0.004, 0.09)	0.0320	0.48	(0.06, 0.91)	0.0275	0.16	(0.02, 0.30)	0.0222
With history of DM (n = 91)
PM10	0.43	(0.15, 0.70)	0.0030	2.27	(0.91, 3.64)	0.0016	1.15	(0.63, 1.66)	< 0.0001
O3	0.68	(0.28, 1.08)	0.0012	2.78	(0.79, 4.78)	0.0077	1.22	(0.44, 2.00)	0.0028
NO2	0.55	(0.27, 0.83)	0.0003	2.78	(1.40, 4.17)	0.0002	1.22	(0.68, 1.75)	< 0.0001
p-Values obtained after weighting follow-up observations in the single-pollutant model of PM10, O3, and NO2 on lag day 4, lag day 5, and lag day 7, respectively. Changes in glucose, insulin, and HOMA indices by an IQR-change of PM10 (20.8 µg/m3), O3 (15.1 ppb), and NO2 (10.8 ppb) were obtained after adjusting for age, sex, BMI, cotinine level, and outdoor temperature and dew point of the day.															

**Figure 2:**
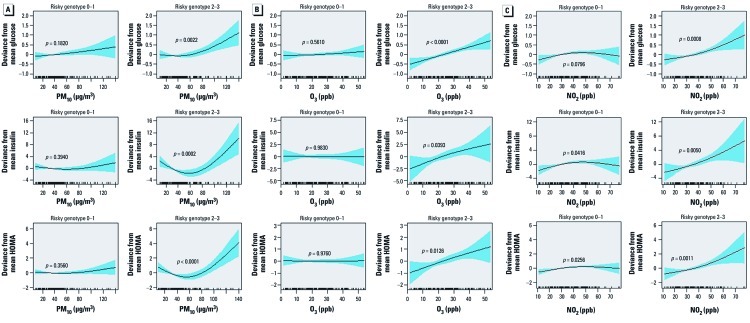
Penalized regression spline of exposure to PM_10_ (*A*), O_3_ (*B*), and NO_2_ (*C*) on lag day 4, lag day 5, and lag day 7, respectively, on glucose (top), insulin (center), and HOMA (bottom) indices by 0–1 (left) or 2–3 (right) risky genotypes. Solid lines, spline curve; shaded area, 95% CI. The curves are adjusted for age, sex, BMI, cotinine level, and outdoor temperature and dew point of the day.

We estimated changes in fasting glucose, insulin, and HOMA associated with IQR increases in PM_10_, O_3_, and NO_2_ by genotype (Table 4). Associations were stronger in participants with *GSTM1*-null, *GSTT1*-null, or *GSTP1* AG or GG genotypes compared with those with *GSTM1* present, *GSTT1* present, or *GSTP1* AA genotypes, although the interaction *p*-values were not always statistically significant. We also estimated associations according to the number of “at-risk” genotypes (defined as *GSTM1*-null, *GSTT1*-null, and *GSTP1* AG or GG genotypes) using linear mixed models or generalized additive mixed models, and found stronger associations between air pollutants and IR indices among those with 2–3 at-risk genotypes compared with only 0–1 and nonlinear association, particularly when those with 2–3 risky genotypes were exposed to PM_10_ (Table 4, Figure 2).

**Table 4:** Associations of PM_10_, O_3_, and NO_2_ with fasting glucose, insulin, and HOMA indices by *GSTM1*, *GSTT1*, and *GSTP1* genotypes.

Gene/genotype		Pollutant	*n*	Glucose						Insulin						HOMA					
				Estimate (95% CI)		*p*-Value	*p* for interaction	Estimate (95% CI)		*p*-Value	*p* for interaction	Estimate (95% CI)		*p*-Value	*p* for interaction
GSTM1	
Present	PM10	225	0.04	(–0.04, 0.13)	0.3019	0.0459	0.19	(–0.25, 0.64)	0.3990	0.6201	0.12	(–0.04, 0.28)	0.1488	0.4944
O3	225	0.04	(–0.10, 0.18)	0.6027	0.0112	0.19	(–0.55, 0.93)	0.6140	0.2934	0.08	(–0.18, 0.34)	0.5552	0.1647
NO2	225	0.06	(–0.02, 0.15)	0.1579	0.2187	0.14	(–0.30, 0.58)	0.5278	0.0054	0.10	(–0.06, 0.25)	0.2308	0.0137
Null	PM10	299	0.17	(0.09, 0.25)	0.0001	0.33	(–0.35, 1.02)	0.3430	0.20	(–0.03, 0.43)	0.0894
O3	299	0.30	(0.17, 0.42)	< 0.0001	1.14	(0.08, 2.19)	0.0351	0.47	(0.11, 0.83)	0.0102
NO2	299	0.14	(0.06, 0.22)	0.0006	1.27	(0.59, 1.94)	0.0003	0.45	(0.22, 0.68)	0.0002
GSTT1	
Present	PM10	254	0.05	(–0.03, 0.12)	0.2449	0.1166	0.17	(–0.41, 0.75)	0.5649	0.8711	0.06	(–0.10, 0.22)	0.4812	0.3609
O3	254	0.08	(–0.03, 0.19)	0.1687	0.0151	0.59	(–0.29, 1.47)	0.1875	0.8825	0.15	(–0.09, 0.40)	0.2231	0.4358
NO2	254	0.02	(–0.05, 0.08)	0.6524	0.0020	0.44	(–0.07, 0.96)	0.0908	0.0463	0.11	(–0.03, 0.25)	0.1338	0.0028
Null	PM10	270	0.15	(0.07, 0.24)	0.0008	0.21	(–0.41, 0.83)	0.5030	0.20	(–0.03, 0.43)	0.0926
O3	270	0.29	(0.15, 0.44)	0.0001	0.77	(–0.27, 1.81)	0.1490	0.42	(0.03, 0.81)	0.0360
NO2	270	0.21	(0.12, 0.31)	< 0.0001	1.08	(0.41, 1.76)	0.0019	0.49	(0.24, 0.75)	0.0002
GSTP1	
AA	PM10	345	0.09	(0.03, 0.15)	0.0052	0.9598	0.06	(–0.47, 0.59)	0.8326	0.7265	0.05	(–0.13, 0.23)	0.5758	0.3947
O3	345	0.08	(–0.02, 0.17)	0.1029	0.0006	0.15	(–0.07, 0.97)	0.7215	0.0205	0.06	(–0.22, 0.34)	0.6762	0.0036
NO2	345	0.08	(0.02, 0.14)	0.0077	0.3620	0.75	(0.24, 1.26)	0.0042	0.3848	0.26	(0.09, 0.43)	0.0034	0.4972
AG or GG	PM10	179	0.12	(0.001, 0.24)	0.0503	0.47	(–0.26, 1.20)	0.2099	0.29	(0.04, 0.54)	0.0250
O3	179	0.40	(0.20, 0.60)	0.0001	1.79	(0.58, 3.01)	0.0044	0.74	(0.32, 1.17)	0.0007
NO2	179	0.14	(0.02, 0.27)	0.0230	0.70	(–0.05, 1.44)	0.0695	0.30	(0.04, 0.56)	0.0235
At-risk genotypea		
0–1	PM10	282	0.08	(0.005, 0.15)	0.0382	0.6077	0.04	(–0.37, 0.45)	0.8377	0.3584	0.06	(–0.07, 0.18)	0.3853	0.2277
O3	282	0.05	(–0.06, 0.17)	0.3625	0.0003	–0.06	(–0.69, 0.57)	0.8633	0.0860	–0.01	(–0.20, 0.19)	0.9379	0.0244
NO2	282	0.05	(–0.02, 0.12)	0.1832	0.0358	0.32	(–0.05, 0.69)	0.0943	0.0135	0.11	(–0.001, 0.23)	0.0528	0.0030
2–3	PM10	242	0.13	(0.03, 0.22)	0.0080	0.38	(–0.39, 1.15)	0.3388	0.23	(–0.04, 0.59)	0.1017
O3	242	0.35	(0.20, 0.50)	< 0.0001	1.47	(0.19, 2.74)	0.0253	0.61	(0.16, 1.06)	0.0089
NO2	242	0.19	(0.09, 0.29)	0.0003	1.28	(0.45, 2.11)	0.0027	0.51	(0.22, 0.81)	0.0006
p-Values obtained after weighting follow-up observations in the single-pollutant model of PM10, O3, and NO2 on lag day 4, lag day 5, and lag day 7, respectively. Changes in glucose, insulin, and HOMA indices by an IQR-change of PM10 (20.8 µg/m3), O3 (15.1 ppb), and NO2 (10.8 ppb) were obtained after adjusting for age, sex, BMI, cotinine level, and outdoor temperature and dew point of the day. aNumber of risky genotypes.																									

We compared estimates and statistical significance before and after weighting follow-up observations to evaluate potential selection bias, and before and after adjusting for hypertension and hyperlipidemia to evaluate possible confounding due to preexisting conditions that could be related to air pollution exposure and IR, and found no difference (data not shown).

## Discussion

Our findings suggest associations of the air pollutants PM_10_, O_3_, and NO_2_ with fasting glucose, insulin, and HOMA index values in the elderly and that participants with *GSTM1*-null, *GSTT1*-null, and *GSTP1* AG or GG genotypes may be more susceptible to effects of air pollution on IR.

Several studies have explored potential effects of air pollution on DM. A case–control study reported that exposure to PM_10_ was significantly higher for 61 children diagnosed with DM compared with 39 controls (Hathout et al. 2002). Brook et al. (2008) studied the relationship between DM and exposures to traffic pollution using NO_2_ measurements, and they reported a statistically significant increase of 17% in DM with a 4-ppb increase in NO_2_ exposure among women, but not among men. DM-related mortality has also been associated with PM, SO_2_, and NO_2_ exposures (Kan et al. 2004; Maynard et al. 2007; Ostro et al. 2006). Several studies have reported positive associations between PM ≤ 2.5 µm in diameter (PM_2.5_) and insulin or other IR-related indices such as whole-body IR, systemic inflammation, and visceral adiposity (Kelishadi et al. 2009; Sun et al. 2009) and evidence of effect modification by impaired glucose homeostasis of associations between PM_10_ and heart rate variability (Whitsel et al. 2009). Because DM and metabolic syndrome are chronic inflammatory states, air pollution, which is known to increase systemic and adipose tissue inflammation (Pope et al. 2004; Sun et al. 2009; van Eeden et al. 2001), could induce or exaggerate IR (Sun et al. 2009). A population-based study of 374 children 10–18 years of age supported the air pollution effect by showing an association of air quality with C-reactive protein and HOMA index values (Kelishadi et al. 2009). Animal studies have also shown that particulate air pollution exposure increases IR, and reactive oxygen species mediate this increased risk of IR (Sun et al. 2009; Xu et al. 2010). We found positive associations of PM_10_, O_3_, and NO_2_ with fasting glucose, insulin, and HOMA indices, indicating that these pollutants may affect the development of DM. In addition, we found differences in the time windows of apparent effects. Ozone appeared to have a more acute effect compared with PM_10_ and NO_2_ (lag days 0–5 vs. 0–10 and 0–8, respectively). However in this study, we did not find evidence of associations between SO_2_ and the IR indices. The main sources of SO_2_ have been known to be combustion in energy and transformation industries, whereas the main source of PM_10_, O_3_, and NO_2_ is road-transport related, although the contribution of different sources varies between and within countries. Therefore PM_10_, O_3_, and NO_2_, but not SO_2_, are likely markers of traffic in the study area.

The majority of the literature to date suggests that DM functions as an effect modifier on the relationship between exposure to air pollution and cardiovascular outcome, as opposed to being a direct consequence of exposure (Zanobetti and Schwartz 2001, 2002). Other researchers have reported stronger associations between air pollution exposure and cardiovascular hospitalizations or emergency room visits among persons with DM compared with those without DM (Peel et al. 2007; Pereira Filho et al. 2008). In our study, associations between PM_10_, O_3_, and NO_2_ and IR indices remained even after excluding subjects with a history of DM although air pollutants had a stronger effect on IR in these subjects. Research has suggested that inflammatory mechanisms exacerbate the impact of air pollution among persons with DM. In our study, a change in glucose level associated with a change of PM_10_, O_3_, and NO_2_ in subjects with a history of DM was larger than in those without a history of DM. Although a comparison between our results and those of previous studies is difficult because of the different outcomes, participants with DM appeared to be more susceptible to the apparent effects of air pollutant exposures on the IR indices than were those without DM.

We therefore hypothesized that insulin signaling and downstream pathways may mediate the effect of air pollution on chronic diseases, including DM. Recently, Sun et al. (2009) demonstrated that ambient PM_2.5_ potentiated the effect of obesity on IR in a diet-induced murine model of obesity. Their results suggest that the previously observed link between PM and DM may be mediated through IR and visceral inflammation due to PM (Brook et al. 2008; Chen and Schwartz 2008). Knuckles and Dreher (2007) studied changes in transcription and translation in rat neonatal cardiomyocyte cultures after an acute exposure to bioavailable constituents of PM_2.5_ oil combustion particles. Genomic alterations observed included changes in insulin/insulin-like growth factor 1 (IGF-1) and phosphatidylinositol-3 (PI3)/serine/threonine specific protein kinase (Akt) signaling, which suggest the involvement of insulin in the response to the particles within cardiomyocytes. Diesel exhaust emissions can activate redox-sensitive transcription factors, including nuclear factor kappa-B (NF-κB) and activator protein 1 (AP1), both of which have been linked to insulin/IGF-1 signaling. The PI3/AKT pathway, which is in part regulated by insulin/IGF-1 signaling, plays a key role in cell cycle progression (Liang and Slingerland 2003), although the detailed mechanisms are still poorly understood.

An individual’s susceptibility to IR conferred by air pollution exposure could vary depending on genetic factors (Minelli et al. 2011). Because air pollution exposure induces oxidative stress, which is known to mediate development of IR, genes modulating oxidative stress are good candidates for investigating gene × air pollution interactions (Minelli et al. 2011). *GSTM1*, *GSTT1*, and *GSTP1* defend against oxidative stress by conjugating reactive oxygen species with glutathione, which detoxifies and eliminates them. O_3_ is a strong oxidant that exerts its action either by direct reaction with target molecules or by generating reactive oxygen species (Romieu et al. 2010). NO_2_ is directly involved in pulmonary inflammation and contributes to reactive oxygen species indirectly by the formation of O_3_ (Romieu et al. 2010). Oxidative stress is also produced by enzymatically catalyzed reactions in target cells by organic chemicals and transition metals bound to the surfaces of PM (Bae et al. 2010). We found that estimated effects of PM_10_, O_3_, and NO_2_ on the IR indices were stronger in participants with *GSTM1*- or *GSTT1*-null genotypes or an AG or GG genotype of *GSTP1* and that associations were stronger in participants with more than one at-risk genotype of *GSTM1*, *GSTT1*, or *GSTP1*. These findings suggest that the capacity to scavenge oxygen free radicals induced by air pollution exposure is different depending on genetic polymorphisms of *GSTM1*, *GSTT1*, and *GSTP1*. Because age-related diseases are increasing as a result of changes in lifestyle and environment and because genetic factors may influence the development of age-related diseases (Perls 2006), gene × environmental interactions should also be considered when studying air pollution–related health effects in the elderly.

The study of genetic susceptibility can clarify air pollution pathophysiological mechanisms and help identify susceptible populations that should be targets of public health interventions. Enhancing antioxidant defenses, such as antioxidant supplementation in susceptible persons, could be a potentially preventive measure (Minelli et al. 2011). In addition, air quality standards based on average effects in the population as a whole should be revised to protect genetically susceptible populations (Minelli et al. 2011).

To the best of our knowledge, this is the first epidemiological study to investigate the role of genetic polymorphisms in the effects of outdoor exposure to air pollutants on IR. However, there were some limitations to this study. We recruited subjects ≥ 60 years of age; if age modifies the effect of air pollution on IR, our results may not be generalizable to younger people. We did not precisely measure each individual’s exposure level to ambient air pollutants; instead, we used monitoring data for the site nearest to their home. Because most of the elderly subjects were not employed, exposure assignment based on the residential address of subjects was reasonable although exposure misclassification cannot be completely ruled out. Moreover, such an error is likely to be nondifferential, which generally shifts the associations toward the null. We also did not adjust for socioeconomic status even though socioeconomic status may be a potentially important confounder of the association between air pollution exposure and DM. Individual exposure to other pollutants or other confounding factors could have biased the results if they had increased concomitantly with the measured air pollution levels; however, the possibility of this scenario is low. Although we adjusted for other potential risk factors for IR—specifically age, sex, smoking, and BMI—concomitant exposure to other pollutants, such as volatile organic chemicals or heavy metals, could also have had some impact on IR.

## Conclusion

Overall, short-term exposure to air pollution was significantly associated with markers of IR in our elderly study population. In addition, participants with *GSTM1*-null, *GSTT1*-null, and *GSTP1* AG or GG genotypes showed stronger associations between IR markers and exposure to air pollution, suggesting genetic susceptibility. These findings shed new light on the relationship among exposure to air pollutants, IR responses, and GST gene polymorphisms.

## Supplemental Material

(143 KB) PDF
