# Supplementary material for: GSTM1, GSTT1, and GSTP1 Polymorphisms and Associations between Air Pollutants and Markers of Insulin Resistance in Elderly Koreans
Source: Environ Health Perspect. 2012 Jun 25;120(10):1378–84. doi: 10.1289/ehp.1104406 (PMC3491923; doi:10.1289/ehp.1104406)
Supplement: (143 KB) PDF [file ehp.1104406.s001.pdf]

Supplemental Material;

GSTM1, *GSTT1*, and *GSTP1* Polymorphisms and Associations between Air Pollutants and Markers of Insulin Resistance in Elderly Koreans

Jin Hee Kim[1] and Yun-Chul Hong[1,2]

[1]Institute of Environmental Medicine, Seoul National University Medical Research Center, Seoul, Republic of Korea; [2]Department of Preventive Medicine, Seoul National University College of Medicine, Seoul, Republic of Korea

Table of Contents

Supplemental Material, Table S1. P2

Supplemental Material, Table S2. P3

Supplemental Material, Table S3. P5

Supplemental Material, Figure S1. P6

Supplemental Material, Figure S2. P7

Supplemental Material, T able S1. Correlation among PM_{10}, SO_2, O_3, and NO_2 exposure levels in the health examination day. Correlation coefficient and *p*-value were presented.

	PM_{10}	SO_2	O_3	NO_2
**PM_{10}**	1	0.61	-0.12	0.65
		<.0001	<.0001	<.0001
**SO_2**		1	-0.30	0.56
			<.0001	<.0001
**O_3**			1	-0.35
				<.0001
**NO_2**				1

Supplemental Material, Table S2. Associations of PM_{10}, SO_2, O_3, and NO_2 with glucose, insulin, and HOMA indices according to lag day.

		Glucose			Insulin			HOMA		
	Lag day	Estimate	95% CI	*p*-Value	Estimate	95% CI	*p*-Value	Estimate	95% CI	*p*-Value
PM_{10}	Lag 0	0.01	-0.05, 0.08	0.6706	-0.01	-0.46, 0.44	0.9586	-0.03	-0.18, 0.13	0.7404
	Lag 1	0.002	-0.07, 0.07	0.9581	0.07	-0.43, 0.57	0.7794	0.04	-0.13, 0.21	0.6560
	Lag 2	0.08	0.004, 0.15	0.0401	0.24	-0.28, 0.77	0.3631	0.14	-0.04, 0.32	0.1270
	Lag 3	0.09	0.02, 0.15	0.0087	0.26	-0.20, 0.73	0.2698	0.12	-0.04, 0.28	0.1472
	Lag 4	0.11	0.05, 0.17	0.0005	0.21	-0.22, 0.64	0.3439	0.14	-0.003, 0.29	0.0549
	Lag 5	0.05	-0.01, 0.10	0.1236	0.11	-0.30, 0.52	0.6082	0.08	-0.07, 0.22	0.2924
	Lag 6	0.06	0.01, 0.11	0.0166	0.17	-0.18, 0.52	0.3490	0.06	-0.06, 0.19	0.2982
	Lag 7	0.07	0.02, 0.12	0.0099	0.02	-0.36, 0.40	0.9264	0.03	-0.10, 0.16	0.6728
	Lag 8	0.10	0.05, 0.16	0.0002	0.06	-0.34, 0.46	0.7758	0.07	-0.07, 0.20	0.3447
	Lag 9	0.02	-0.03, 0.07	0.4418	-0.15	-0.52, 0.22	0.4352	-0.03	-0.16, 0.10	0.6596
	Lag 10	0.05	-0.01, 0.11	0.0866	0.22	-0.20, 0.63	0.3057	0.08	-0.06, 0.22	0.2594
SO_2	Lag 0	-0.05	-0.11, 0.01	0.1314	0.04	-0.40, 0.47	0.8752	-0.03	-0.18, 0.12	0.6829
	Lag 1	-0.04	-0.10, 0.02	0.1825	0.13	-0.30, 0.56	0.5457	0.04	-0.11, 0.18	0.6223
	Lag 2	0.02	-0.04, 0.08	0.5099	0.09	-0.34, 0.53	0.6707	0.08	-0.07, 0.23	0.2801
	Lag 3	0.07	0.01, 0.13	0.0259	0.34	-0.10, 0.78	0.1342	0.15	-0.003, 0.30	0.0552
	Lag 4	0.06	-0.003, 0.12	0.0647	0.30	-0.13, 0.73	0.1689	0.15	0.001, 0.29	0.0490
	Lag 5	-0.002	-0.06, 0.06	0.9614	0.005	-0.42, 0.43	0.9825	0.02	-0.12, 0.17	0.7764
	Lag 6	0.02	-0.04, 0.08	0.4556	0.19	-0.23, 0.60	0.3714	0.06	-0.08, 0.20	0.4029
	Lag 7	0.10	0.03, 0.16	0.0026	0.30	-0.14, 0.74	0.1825	0.13	-0.02, 0.28	0.0907
	Lag 8	0.08	0.02, 0.15	0.0148	0.17	-0.30, 0.64	0.4800	0.09	-0.07, 0.25	0.2860
	Lag 9	0.06	-0.02, 0.13	0.1338	0.29	-0.21, 0.79	0.2540	0.10	-0.07, 0.27	0.2391
	Lag 10	0.04	-0.04, 0.11	0.3469	0.38	-0.14, 0.91	0.1505	0.11	-0.07, 0.29	0.2364
O_3	Lag 0	0.11	-0.01, 0.22	0.0756	-0.05	-0.87, 0.78	0.9138	-0.01	-0.30, 0.27	0.9235
	Lag 1	0.19	0.09, 0.29	0.0002	0.42	-0.31, 1.14	0.2592	0.22	-0.03, 0.47	0.0858
	Lag 2	0.08	-0.02, 0.18	0.1009	0.42	-0.27, 1.12	0.2297	0.17	-0.07, 0.40	0.1677
	Lag 3	0.09	-0.004, 0.19	0.0607	0.36	-0.35, 1.06	0.3196	0.12	-0.12, 0.36	0.3367
	Lag 4	0.11	0.02, 0.21	0.0211	0.78	0.10, 1.46	0.0252	0.25	0.02, 0.48	0.0375
	Lag 5	0.19	0.09, 0.28	0.0001	0.70	0.02, 1.39	0.0439	0.30	0.06, 0.53	0.0139
	Lag 6	0.10	-0.002, 0.20	0.0549	-0.02	-0.76, 0.72	0.9612	-0.02	-0.27, 0.24	0.8948
	Lag 7	0.01	-0.10, 0.11	0.9026	-0.57	-1.31, 0.18	0.1353	-0.22	-0.47, 0.04	0.0966
	Lag 8	0.09	-0.01, 0.19	0.0838	0.30	-0.41, 1.01	0.4102	0.09	-0.15, 0.34	0.4500
	Lag 9	0.07	-0.02, 0.16	0.1480	0.35	-0.31, 1.02	0.2994	0.16	-0.07, 0.39	0.1699
	Lag 10	0.03	-0.06, 0.13	0.5055	0.51	-0.20, 1.22	0.1583	0.15	-0.09, 0.39	0.2224
NO_2	Lag 0	0.03	-0.03, 0.09	0.3734	0.49	0.04, 0.94	0.0342	0.15	0.0002, 0.31	0.0503

Lag 1	0.01	-0.05, 0.08	0.7364	0.33	-0.14, 0.79	0.1675	0.13	-0.03, 0.29	0.1005
Lag 2	0.05	-0.02, 0.12	0.1329	0.47	0.005, 0.94	0.0483	0.20	0.04, 0.36	0.0126
Lag 3	0.07	0.01, 0.13	0.0310	0.23	-0.21, 0.66	0.3090	0.13	-0.02, 0.28	0.0937
Lag 4	0.08	0.02, 0.14	0.0078	0.07	-0.34, 0.47	0.7554	0.09	-0.05, 0.23	0.2154
Lag 5	0.04	-0.02, 0.10	0.1765	0.001	-0.41, 0.41	0.9977	0.03	-0.11, 0.17	0.6619
Lag 6	0.05	-0.01, 0.11	0.1121	0.41	-0.02, 0.84	0.0628	0.15	0.004, 0.30	0.0452
Lag 7	0.11	0.05, 0.17	0.0004	0.71	0.29, 1.14	0.0010	0.28	0.13, 0.42	0.0002
Lag 8	0.08	0.02, 0.14	0.0124	0.35	-0.10, 0.79	0.1307	0.16	0.01, 0.32	0.0354
Lag 9	0.03	-0.03, 0.10	0.3184	0.34	-0.13, 0.81	0.1543	0.08	-0.08, 0.25	0.3039
Lag 10	-0.02	-0.08, 0.04	0.5795	0.26	-0.18, 0.70	0.2508	0.03	-0.12, 0.18	0.6573

Changes in glucose, insulin, and HOMA indices by an IQR-change of PM_{10} (20.8 μg/m^3), SO_2 (1.6 ppb), O_3 (15.1 ppb), and NO_2 (10.8 ppb) were estimated in linear mixed-effect models after weighting follow-up observations and adjusting for age, sex, BMI, cotinine level, and outdoor temperature and dew point in the day. CI, Confidence Interval.

Supplemental Material, Table S3. Associations of PM_{10}, O_3, and NO_2 with glucose, insulin, and HOMA indices in multiple pollutant models.

	Glucose			Insulin			HOMA		
	Estimate	95% CI	*p*-Value	Estimate	95% CI	*p*-Value	Estimate	95% CI	*p*-Value
Two pollutant model									
PM_{10}	0.08	0.01, 0.14	0.0178	0.07	-0.38, 0.52	0.7579	0.09	-0.06, 0.25	0.2345
O_3	0.15	0.05, 0.25	0.0036	0.67	-0.06, 1.39	0.0711	0.25	-0.001, 0.49	0.0518
PM_{10}	0.08	0.02, 0.15	0.0074	0.03	-0.41, 0.47	0.8955	0.08	-0.07, 0.23	0.2989
NO_2	0.08	0.02, 0.15	0.0068	0.71	0.27, 1.14	0.0016	0.26	0.11, 0.41	0.0007
O_3	0.16	0.06, 0.25	0.0011	0.49	-0.20, 1.19	0.1649	0.21	-0.02, 0.45	0.0787
NO_2	0.09	0.03, 0.15	0.0049	0.65	0.22, 1.08	0.0031	0.25	0.10, 0.40	0.0009
Three pollutant model									
PM_{10}	0.06	-0.005, 0.12	0.0695	-0.07	-0.53, 0.39	0.7758	0.04	-0.11, 0.20	0.5799
O_3	0.13	0.03, 0.23	0.0094	0.52	-0.20, 1.25	0.1582	0.19	-0.06, 0.44	0.1280
NO_2	0.07	0.01, 0.14	0.0179	0.67	0.23, 1.11	0.0032	0.24	0.09, 0.39	0.0016

p-Values obtained after weighting follow-up observations of PM_{10}, O_3, and NO_2 on lag day 4, lag day 5, and lag day 7, respectively. Changes in glucose, insulin, and HOMA indices by an IQR-change of PM_{10} (20.8 $\mu g/m^3$), O_3 (15.1 ppb), and NO_2 (10.8 ppb) were estimated after adjustment for age, sex, BMI, cotinine level, and outdoor temperature and dew point in the day. CI, Confidence Interval.

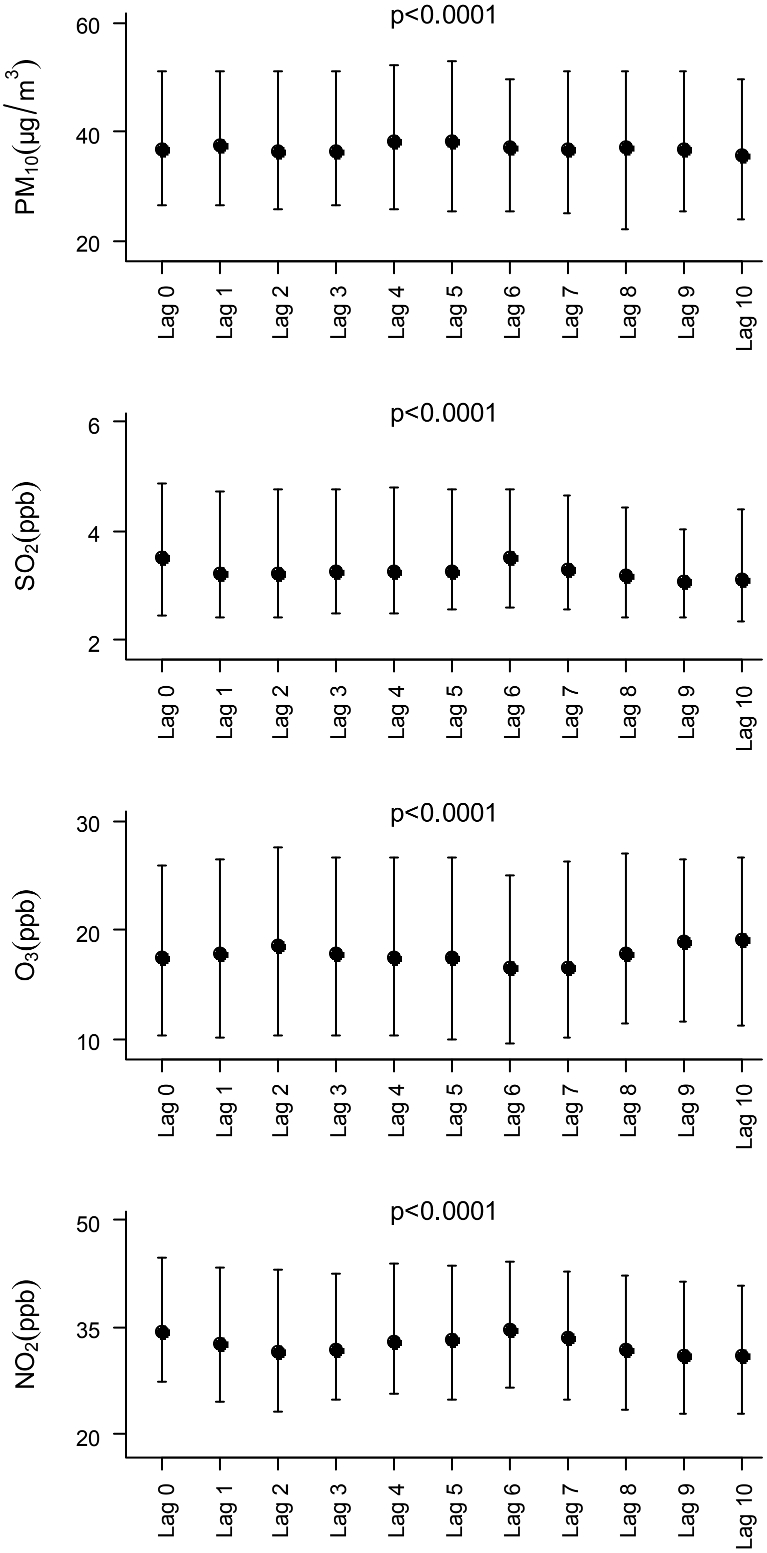

Supplemental Material, Figur e S1. Variation of air pollutant levels over 11 days including the health examination day. Median values of each pollutants and ranges between 25th and 75th percentiles were presented.

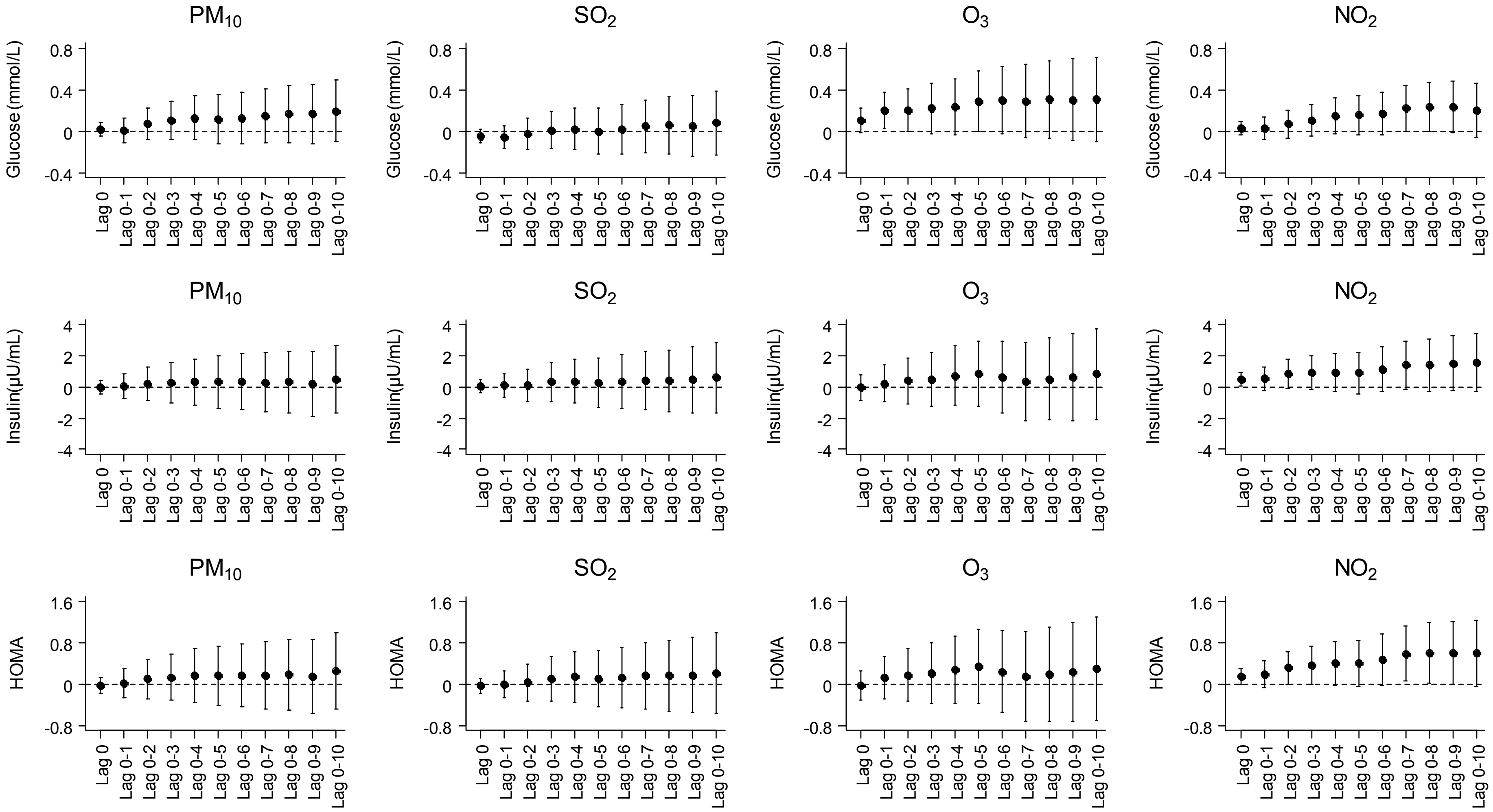

Supplemental Material, Figure S2. Associations of PM_{10}, SO_2, O_3, and NO_2 with glucose, insulin, and HOMA indices by distributed lag days in total subjects. Changes in glucose, insulin, and HOMA indices by an IQR-change of PM_{10} (20.8 μg/m^3), SO_2 (1.6 ppb), O_3 (15.1 ppb), and NO_2 (10.8 ppb) were estimated after adjustment for age, sex, BMI, cotinine level, and outdoor temperature and dew point in the day.
